# The Hidden Players in Multiple Sclerosis Nutrition: A Narrative Review on the Influence of Vitamins, Polyphenols, Salt, and Essential Metals on Disease and Gut Microbiota

**DOI:** 10.3390/nu18010148

**Published:** 2026-01-01

**Authors:** Rachele Rosso, Eleonora Virgilio, Matteo Bronzini, Simona Rolla, Alessandro Maglione, Marinella Clerico

**Affiliations:** 1 Department of Clinical and Biological Sciences, University of Turin, 10043 Orbassano, Italy; rachele.rosso@unito.it (R.R.); eleonora.virgilio@unito.it (E.V.); alessandro.maglione@unito.it (A.M.); marinella.clerico@unito.it (M.C.); 2 San Luigi Gonzaga University Hospital, 10043 Orbassano, Italy; bronzini.matteo@gmail.com; 3 Department of Computer Science, University of Turin, 10149 Torino, Italy

**Keywords:** micronutrients, vitamins, essential metals, polyphenols, salt, multiple sclerosis, experimental autoimmune encephalomyelitis, gut microbiota

## Abstract

Multiple sclerosis (MS) is a chronic neuroinflammatory and autoimmune disorder of the central nervous system (CNS) whose cause remains unknown. Disease-modifying therapies (DMTs) are the current standard of care, yet growing evidence highlights the importance of complementary lifestyle-based interventions, including nutrition, in modulating disease activity. Given the influence of diet on immune function, several studies have examined its effects in MS, with particular attention to specific dietary patterns and macronutrients. However, fewer studies have focused on micronutrients, bioactive compounds, and minerals and their influence in MS. In this narrative review, we report the latest evidence on micronutrients such as vitamins and essential metals, along with polyphenols and minerals like salt, in both experimental autoimmune encephalomyelitis (EAE) and MS. We also discuss how these dietary components may influence the gut microbiota, which is considered a contributor to disease onset due to its interaction with the immune system in the gut–brain axis. While findings for vitamins B, C, E, and K remain heterogeneous, vitamins A and D show the most consistent immunological and clinical effects, with immunomodulatory, antioxidative, and neuroprotective effects in both EAE and MS. Polyphenols also display anti-inflammatory and neuroprotective properties in EAE and, to a lesser extent, in clinical studies. Lastly, evidence suggests the importance of balanced salt intake and adequate levels of essential metals, as dysregulation may contribute to comorbidities or enhance inflammatory pathways relevant to MS. Although only a limited number of studies have explored these aspects, the gut microbiota appears to be differentially affected by these dietary factors. Overall, advancing our understanding of how these components interact with immune and microbial pathways may support the development of personalized nutritional strategies to complement current therapies and improve patient outcomes.

## 1. Introduction

Currently, approximately 2.9 million people worldwide are living with multiple sclerosis (MS), a chronic autoimmune and neuroinflammatory disease that affects the central nervous system (CNS). MS is characterized by inflammation, demyelination, gliosis, and neuronal loss, and can lead to severe disability in affected individuals. Common clinical manifestations include visual impairment, focal weakness, cognitive dysfunction, and fatigue, together with typical magnetic resonance imaging (MRI) features. The average age at diagnosis ranges between 20 and 50 years, and women are affected more frequently than men [1,2,3]. The immune cells primarily involved in MS pathogenesis are T helper (Th) cells, particularly Th1 and Th17 subsets, which produce interferon-γ (IFN-γ) and interleukin-17 (IL-17), respectively [4]. B cells also play a crucial role, mainly through the production of autoantibodies directed against myelin self-proteins such as myelin basic protein (MBP) and proteolipid protein (PLP) [5,6,7]. Furthermore, regulatory T cells (Tregs) are implicated in MS pathology, as a reduction in their suppressive activity has been observed in people with MS (pwMS) [8]. Neuroinflammation in MS is characterized by the infiltration of these immune cells from the periphery into the CNS, facilitated by disruption of the blood–brain barrier (BBB) and activation of resident glial cells. This inflammatory process then becomes chronic when tissue repair mechanisms fail. Two main types of inflammation can be distinguished in MS: focal inflammation, defined by T and B lymphocyte infiltration associated with BBB breakdown and the subsequent formation of demyelinating plaques; and compartmentalized inflammation, in which T and B cells accumulate in the meninges and perivascular spaces without BBB disruption [4].

Several clinical phenotypes of MS have been identified, including relapsing–remitting MS (RRMS), primary progressive MS (PPMS), and secondary progressive MS (SPMS). RRMS accounts for approximately 85% of cases and is characterized by alternating periods of relapse and remission, meaning periods of acute phases with neurological dysfunction followed by periods of clinical stability and absence of symptoms. PPMS occurs in about 10–20% of patients and is distinguished by a continuous progression of neurological disability from disease onset, without the initial relapsing–remitting phase observed in RRMS. Most RRMS patients eventually develop SPMS. SPMS typically exhibits a less uniform disease course, with periods of gradual progression with relapses and occasional phases of clinical stability [9]. Additionally, it is defined as a clinically isolated syndrome (CIS), the first clinical onset of MS, characterized by acute and subacute episodes [10]. The 2014 Lublin classification has updated these definitions of MS phenotypes. Although the traditional subdivision into RRMS, PPMS, and SPMS is retained, the revised scheme introduces an additional descriptor, active or non-active, based on the presence or absence of new clinical relapses or MRI activity [11]. Lastly, it is defined as pediatric MS (PMS), the disease that develops in children and adolescents under the age of 18. This form represents approximately 3–10% of all MS cases and differs from adult-onset MS in several ways. Indeed, PMS generally experience a high relapse rate, with initially a higher ability to recover, but early cognitive impairment and transition to SPMS compared to adults [12]. MS may also manifest after the age of 50 (late-onset MS, LOMS), with reported prevalence ranging from about 1.1% to over 21%, depending on the age cut-offs used in the literature. Patients with LOMS tend to shift to SP stages more frequently, indicating that age-related physiological processes may contribute to a more pronounced and chronic axonal degeneration [13].

Currently, the exact cause of MS remains unknown, but a combination of genetic and environmental factors contributes to disease susceptibility and onset. Genetic predisposition plays a significant role, particularly variations within the human leukocyte antigen (HLA) region located on chromosome 6, and specifically the HLA-DRB1 gene alleles, which have been strongly associated with increased MS risk [14]. Among environmental factors, pollution, stress, smoking, and infections, especially Epstein-Barr Virus (EBV), have been considered as potential contributors to MS development [15]. Vitamin D has also emerged as a relevant factor. Epidemiological evidence shows that MS prevalence is higher in northern regions and gradually decreases toward southern latitudes. This geographic gradient has been attributed to differences in exposure to ultraviolet B (UVB) radiation, which influences cutaneous Vitamin D synthesis [16]. Finally, two closely related factors believed to influence MS development are nutrition and the gut microbiota. Dietary components can exert either beneficial or detrimental effects on immune regulation, depending on their nutritional properties. This occurs through modulation of the gut microbiota, the community of microorganisms that lives in the intestine, which interacts with the gut-associated lymphoid tissue (GALT) rich in immune cells [17]. Consequently, different nutrients can promote the growth of specific microbial populations that in turn exert pro- or anti-inflammatory effects on the immune system. These diet–microbiota–immune interactions have been documented both in healthy individuals and in patients with various diseases [18,19,20], underscoring the potential importance of dietary modulation as an adjunct strategy for the prevention and management of diseases as MS.

Several disease-modifying therapies (DMTs) are currently used for MS treatment. These therapies can be categorized by route of administration, by mechanism of action, and by efficacy on neuroinflammation and neurodegeneration. Indeed, DMTs may be injectable, oral, or administered intravenously, and their mechanisms include selective immunomodulation, inhibition of lymphocyte proliferation or activation, lymphocyte trafficking blockade, B-cell depletion, and broad immune cell depletion [21]. Since the underlying cause of MS remains unknown, DMTs prevent new clinical relapse and radiological activity, modify the disease course, reduce symptoms, and help patients better manage and live with the condition. Currently their efficacy in preventing progression independent from relapse activity is limited [22]. In this context, complementary lifestyle interventions have also been associated with clinical benefits. For example, adopting a healthy and balanced diet, tailored to the patient’s individual needs, may support overall treatment outcomes alongside pharmacological therapy [17].

Given the key role of diet and its effects on the immune system, several studies have investigated the impact of nutrition in MS, primarily focusing on specific dietary patterns or the effects of macronutrients on disease course [23]. In contrast, relatively few studies have explored the role of micronutrients, bioactive compounds, and minerals and their potential influence on overall health in MS. In this review, we present the most recent evidence regarding the role of micronutrients, such as vitamins and essential metals, polyphenols, and minerals such as salt in experimental autoimmune encephalomyelitis (EAE) and MS. Additionally, we highlight existing literature on the effects of these elements on the gut microbiota, with the final aim of contributing to a clearer understanding of the potential benefits that dietary interventions may provide in the management of MS.

## 2. Methods

This narrative review includes data from scientific papers collected across several databases, including PubMed, Google Scholar, ScienceDirect, Scopus, and Web of Science. The literature search was conducted to collect evidence on the role of micronutrients and dietary supplements, such as vitamins, polyphenols, salt, and metals, in modulating EAE and MS pathology, also accounting for their impact on gut microbiota composition and function. Specific keywords such as “micronutrients”, “vitamins”, “polyphenols”, “salt”, and “metals” were used for the research, which were combined with terms like “experimental autoimmune encephalomyelitis”, “multiple sclerosis”, and “gut microbiota”, using Boolean operators (AND, OR) to refine the results. Article selection was performed by screening the articles identified through this search process, initially by reviewing the abstracts to assess relevance to the research question, followed by full-text evaluation to extract informations relevant to the review. The final literature review was completed in December 2025 and included studies relevant to the objective of the review, with no time limitation, providing an updated overview of the literature. The inclusion criteria comprised original research articles and review papers relevant to the topic of this work, including animal and in vitro model and human studies, such as preclinical and clinical trials, pilot studies, randomized controlled trials, case–control, cross-sectional, and cohort studies, observational, retrospective, and prospective studies, as well as meta-analyses, systematic reviews, and narrative reviews, evaluating the association between micronutrients and dietary supplements and EAE and MS. Studies addressing subjects consistent with the aims of the review were included, whereas those not meeting these criteria and not published in English language were excluded. Whenever conflicting findings on a given topic were identified, all available data were considered, incorporated into the text, and critically discussed in light of the different conditions under which the results were obtained, to provide an understanding of the current knowledge. As this is a narrative review, detailed documentation of the literature searches across specific platforms is not required.

## 3. Vitamins

Vitamins are a group of organic compounds essential for normal growth and nutrition which must be introduced through the diet because they cannot be synthesized endogenously. Vitamins are categorized based on their types and properties, and the evidence regarding their relationship with MS is discussed in detail below and summarized in Table 1.

### 3.1. Vitamin A

Vitamin A, also called retinol, can be obtained through the diet in various forms, such as all-trans retinol, retinyl esters, or β-carotene. It is mainly present in oily fish, liver, eggs, cheese, and butter; following adsorption, it is metabolized into all-trans retinoic acid (RA), which represents the biologically active form of Vitamin A [75,76]. Starting from the observation that CD103^+^ gut-associated dendritic cells (DCs) are capable of synthesizing RA, it is known that RA promotes the differentiation of forkhead box P3 (Foxp3^+^) regulatory T cells and Th2 cells, as well as the induction of IgA-secreting antibody-secreting cells and the inhibition of intestinal Th17 cells [77,78,79], mechanisms involved in MS pathogenesis. Moreover, RA is reported to increase the production of IL-10 by B cells in pwMS [26].

Several studies reported a relation between RA and EAE. Indeed, RA appears to be able to suppress EAE [24], and co-supplementation of vitamins A and C was demonstrated to mitigate neurological severity and EAE disease progression [25].

In pwMS, retinol levels have been reported to be lower than in controls or people without non-inflammatory neurological diseases [54,80]. Moreover, increased serum retinol levels appear to correlate with a reduced risk of developing gadolinium-enhancing T1 lesions, T2 lesions, and active lesions, and its concentrations have been shown to predict the appearance of new gadolinium-enhancing T1 lesions and new T2 lesions up to six months in advance [27]. All this evidence suggests a role of Vitamin A deficiency in MS disease activity and progression.

In parallel, RA supplementation has shown beneficial effects on the health of pwMS. Vitamin A indeed was reported to improve MS functional composite (MSFC) in RRMS patients, even if no significant changes were observed for the Expanded Disability Status Scale (EDSS), brain lesions, and relapse rate [28]. Furthermore, Vitamin A supplementation improved fatigue and depression and psychiatric outcomes [29]. On the contrary, focusing on the Vitamin A that derives from carotenoids, current evidence does not support the hypothesis that dietary intake of carotenoids reduces the risk of developing MS in women [56].

### 3.2. Vitamins B

#### 3.2.1. Vitamin B1

Moving to the groups of Vitamin B, Vitamin B1, also known as thiamine, is a coenzyme essential for the metabolism of carbohydrates, fats, and proteins. It is mainly found in whole grains, meat, and fish [81]. Among its various functions, it plays a crucial role in supporting the functionality of the peripheral and CNS by participating in neurotransmitter synthesis.

Its deficiency has been associated with mitochondrial dysfunction, accumulation of lactate and pyruvate, and severe neurological complications, including neuropathy [82].

In EAE, thiamine deficiency exacerbates disease progression by accelerating its onset and promoting microglial activation. This is accompanied by a marked increase in Th1 and Th17 cell infiltration in the spinal cord, thereby amplifying the inflammatory response [30]. In pwMS, dietary intake of Vitamin B1 was negatively correlated with depression levels, highlighting the importance of adequate nutritional support in the management of this condition [31]. At the same time, thiamine supplementation has been observed to provide beneficial effects in reducing fatigue [32].

#### 3.2.2. Vitamin B2

Vitamin B2, also known as riboflavin, is a vitamin obtained from several foods, such as eggs, organ meats (mainly kidneys and liver), lean meats, and milk, which is involved in numerous redox reactions, making it essential for the proper functioning of the nervous, cardiovascular, endocrine, and immune systems [83,84]. In EAE, administration of riboflavin led to beneficial effects on neurological disability mediated by Brain-Derived Neurotrophic Factor (BDNF) and IL-6 and displayed synergistic effects with interferon beta-1a in the EAE process [34,35]. However, a study reported that pwMS who received 10 mg/day of riboflavin supplementation orally for 6 months did not improve their EDSS [36].

#### 3.2.3. Vitamin B3

Niacin, the common name for Vitamin B3, is instead a water-soluble vitamin that could be present or added in food such as poultry, beef, fish, nuts, legumes, and grains and that can be used as dietary supplement [85]. It was reported that Niaspan, a prolonged release form of Niacin, increased the neurological functional recovery compared to the controls in EAE, as well as reduced the inflammatory infiltrates. Also Niaspan promoted an increase in oligodendrogenesis and axonal regeneration [37]. However, a recent study conducted on EAE model found that niacin did not affect EAE clinical score and did not ameliorate neuropathology. Also, even if niacin enhanced phagocytosis by macrophages in culture, it was not able to reduce T cell proliferation [38]. So far, there are no studies in the scientific literature regarding the effect of niacin supplementation in MS.

#### 3.2.4. Vitamin B5

Pantothenic acid or Vitamin B5 can be found in small amount in all the foods, and its main function is the synthesis of co-enzyme A and acyl carrier protein, which are involved in fatty acid synthesis and degradation, transfer of acetyl and acyl groups, together with other anabolic and catabolic processes [86]. Vitamin B5 deficiency is rare and observed only in extreme cases of starvation or malnutrition [86]. In EAE, pantothenic acid was reported to ameliorate Th17-associated autoimmune disorders [39]. Regarding the current knowledge on the relationship between Vitamin B5 and MS, two different studies have reported conflicting results. One study found that pantothenic acid, along with other metabolites, was more elevated in the serum of MS patients than in controls [40]. In contrast, a second study reported that serum levels of Vitamin B5 were significantly lower in MS patients than in healthy controls [41]. Therefore, further research is needed to better understand the role of this vitamin in relation to MS.

#### 3.2.5. Vitamin B6

Vitamin B6, also known as pyridoxine, is present in many foods, particularly in fish, beef liver, and other meats, as well as in fruits, vegetables, and potatoes, or it can be taken as a dietary supplement [87]. It is known that this vitamin, in its coenzyme form, is involved in numerous physiological functions, primarily metabolic processes. In addition to its role in neurotransmitter synthesis, it also contributes to the development and functioning of the immune system [87]. Vitamin B6 has been shown to reduce the accumulation of sphingosine-1-phosphate, and its supplementation was found to prevent the development of EAE, highlighting the anti-inflammatory role of the vitamin in the MS model [42]. Additionally, another study reported that low levels of Vitamin B6 were associated with higher EDSS scores in MS [43].

#### 3.2.6. Vitamin B7

Vitamin B7, also known as biotin, is a nutrient naturally found in food such as organ meats, eggs, fish, meat, seeds, nuts, and certain vegetables [88]. This vitamin functions as an essential cofactor involved in the metabolism of fatty acids, glucose, and amino acids, as well as in histone modification, gene regulation, and cell signaling [88]. One of the first studies investigating the impact of biotin in the EAE model reported that mice were susceptible to the influence of dietary Vitamin B7, as its deficiency led to, among other effects, a marked reduction in thymus size and cellularity, as well as a depressed immune response [44]. Instead, a meta-analysis conducted on three studies on the use of high-dose biotin in pwMS revealed the potential benefit of biotin supplementation for a duration of 12 to 15 months, particularly in terms of improvement in 25-foot walk time [45,89,90,91]. Another study, also conducted on pwMS, further showed that high-dose biotin supplementation had an impact on disability and disease progression in these patients [46].

#### 3.2.7. Vitamin B9 and B12

Vitamin B9 and Vitamin B12 are biologically interconnected vitamins, as a deficiency in the former often leads to a secondary deficiency in the latter. Vitamin B12, also known as cobalamin, is a nutrient found in foods of animal origin and plays a critical role in the development, myelination, and function of the CNS, and it is also essential for red blood cell formation and the synthesis of nucleic acids [92]. Vitamin B9, or folate, is another nutrient obtained from the diet, especially from dark green leafy vegetables, fruits and fruit juices, nuts, beans, peas, seafood, eggs, dairy products, meat, poultry, and grains [92]. It acts as a crucial coenzyme or cosubstrate in nucleic acid synthesis and in the metabolism of amino acids [92]. In EAE, it was reported that the administration of IFN-beta therapy together with B12 led to clinical motorial improvement and to reduced astrocytosis and demyelination, describing the use of Vitamin B12 as a promising support for MS treatment [49].

A meta-analysis conducted in 2022 involving 16 studies found no significant differences in folate and cobalamin concentrations between pwMS and healthy controls. These findings suggest that the levels of these two vitamins do not play a major role in the physical function associated with the disease [47]. Similar results had already been reported in previous meta-analyses, which also did not detect significant differences in folate or cobalamin levels between pwMS and healthy individuals [93,94]. At the same time, according to another study, Vitamin B12 deficiency was also not associated with MS [50]. However, another study reported that pwMS showed lower levels of B12 both in the serum and in the cerebrospinal fluid, and that these lower levels of cobalamin have been associated with earlier age of MS onset [48,51,52]. Conversely, one of these studies found that pwMS receiving cobalamin and folate supplementation significantly improved both the mental and physical fields of quality of life [48].

### 3.3. Vitamin C

Vitamin C, also known as L-ascorbic acid, is an essential vitamin found mainly in fruits and vegetables, particularly in oranges, kiwifruit, strawberries, broccoli and red and green peppers [95]. It exhibits significant antioxidant activity by limiting damage caused by free radicals, and plays a crucial role in the biosynthesis of certain neurotransmitters and in immune function [95]. In mice, administration of ascorbic acid did not affect the clinical course of pre-existing EAE and also had only moderate effects on the development of clinical symptoms, and failed to prevent the opening of the blood–CNS barrier [53]. Moreover, supplementation with Vitamin C in combination with Vitamin A was able to mitigate neurological severity and disease progression in EAE. Specifically, a significant reduction in demyelination, inflammation, immune cell infiltration, and activation of microglia and astrocytes has been observed following co-supplementation with these two vitamins [25]. In MS, it has been reported that serum ascorbic acid levels were significantly lower in pwMS during a relapse compared to healthy controls [54]. This finding was further confirmed by another study conducted on an Egyptian population, in which Vitamin C levels in pwMS were significantly lower than those in controls. Additionally, patients with infratentorial lesions exhibited markedly lower ascorbic acid levels than those without such lesions [55]. At the same time, a study investigating the impact of Vitamin C supplementation on the risk of developing MS reported that the intake of Vitamin C, as well as Vitamin E and multivitamin supplements, did not reduce the risk of MS onset [56].

### 3.4. Vitamin D

Vitamin D, also known as calciferol, is certainly one of the most widely discussed vitamins in relation to its role in MS. It is found in only a few foods but is also produced endogenously when ultraviolet (UV) rays from sunlight reach the skin and stimulate Vitamin D synthesis [96]. Within the body, it plays several important roles, including maintaining adequate calcium and phosphate levels, as well as contributing to the reduction of inflammation and the modulation of immune and neuromuscular function [96]. Moreover, the actions of Vitamin D on neuronal and immune cells within the central nervous system are particularly intriguing, as they support neuron survival by lowering proinflammatory cytokine levels and enhancing the production of neurotrophic factors [63]. The interest in the potential role of Vitamin D in MS arises from several studies suggesting both a link in the development of the disease and a prognostic role [63]. A lower prevalence of MS in countries with higher exposure to UV radiation and in those with greater consumption of Vitamin D-rich oily fish was observed [16]. Starting from the mice model of MS, one study revealed that both Vitamin D and the Vitamin D receptor are required for EAE development [57]. Conversely, another study reported that Vitamin D3, the most physiologically relevant form of Vitamin D, is capable of inhibiting the differentiation and migration of Treg and Th17 cells, thereby protecting against EAE development. These findings suggest that Vitamin D3 may represent a potential preventive therapeutic strategy for Th17-mediated autoimmune diseases [58]. Although much of the research has focused on the general role of Vitamin D in the development of MS [16], some studies have specifically investigated the effects of Vitamin D obtained through diet or supplementation. A study conducted on 187,000 women found that those with a higher dietary intake of Vitamin D had a 33% lower incidence of MS than those with lower intake. Similarly, women who used Vitamin D supplements had a 41% reduced risk of developing MS compared to non-users [59].

Another study conducted on 25 pwMS revealed that supplementation with Vitamin D3 was able to increase IL-10 levels across all groups analyzed, with a more pronounced effect observed in patients with MS [60]. A meta-analysis published in 2024 compared the results of clinical trials conducted up to 2022 that investigated the effects of Vitamin D3 supplementation on clinical outcomes in pwMS. Analysis of nine studies revealed that Vitamin D3 supplementation did not significantly reduce either EDSS scores or the occurrence of new T2 lesions, even if supplementation may still provide long-term clinical benefits [61]. A randomized clinical trial conducted on 303 patients found that oral high-dose Vitamin D was able to reduce disease activity in CIS and in early RRMS [62]. Finally, lower Vitamin D was linked with worse information processing speed performances in newly diagnosed MS [63] and its supplementation could be beneficial on cognition [64].

### 3.5. Vitamin E

Vitamin E, also known as tocopherol, is a vitamin that exists in eight different chemical forms and is well known for its antioxidant properties, which allow it to inhibit the production of reactive oxygen species (ROS) generated during lipid oxidation. In addition to its antioxidant role, Vitamin E is also involved in immune function and other biological processes [97]. Moreover, Vitamin E is present in several foods, mainly in nuts, seeds, vegetable oils, green leafy vegetables, and fortified cereals [97]. In EAE, TFA-12, a tocopherol derivative, has been shown to promote oligodendrocyte regeneration and remyelination [70]. Furthermore, another study demonstrated that treatment with α-tocopherol can attenuate EAE severity and delay disease progression by suppressing T cell proliferation and the Th1 immune response [71]. In pwMS, serum levels of α-tocopherol have been found to be lower than in healthy controls, whereas no significant difference has been observed in the Vitamin E levels in cerebrospinal fluid [54,98]. Another study revealed that Vitamin E supplementation in a population of pwMS significantly reduced lipid peroxidation in serum and helped preserve telomere length in circulating lymphocytes, which is typically increased in MS [72].

### 3.6. Vitamin K

Vitamin K refers to a group of fat-soluble compounds that share a similar chemical structure, known also as naphthoquinones. A distinctive feature of one of these forms, the menaquinones, is that they are primarily of bacterial origin and can be endogenously produced in the human body. Vitamin K is involved in various physiological processes, acting as a coenzyme for Vitamin K-dependent carboxylase and contributing to the functional activity of prothrombin [99]. Although significantly lower levels of Vitamin K2 have been reported in patients with MS than in healthy controls [74], and its administration in EAE has led to a significant improvement in disease outcomes [73], there is currently no evidence regarding the effects of Vitamin K supplementation in pwMS.

### 3.7. Vitamins and the Gut Microbiota

Vitamins are essential nutrients for the human body, and their deficiency can lead to the onset of diseases or the worsening of pre-existing conditions. Moreover, vitamins also exert effects at the level of the gut microbiota, where they can influence both the abundance and the functionality of microbial populations [100]. A study conducted in an EAE model revealed that treatment with several B vitamins together (B1, B2, B3, B5, B6, and B12) was able to improve the gut dysbiosis typically associated with the disease and to maintain homeostasis [33]. However, most studies on the microbiota in MS have focused on the role of Vitamin D, which remains the most widely discussed vitamin in relation to the pathology. Vitamin D plays an important role in maintaining the integrity of the intestinal barrier [65]. Consequently, its deficiency leads to disruption of this barrier, resulting in dysbiosis, specifically, a reduction in butyrate-producing bacteria [66,67]. A study conducted on pwMS, both treated and untreated with glatiramer acetate, reported that untreated pwMS showed an increase in *Faecalibacterium*, *Coprococcus*, and *Akkermansia* following Vitamin D supplementation compared to the other study groups [68]. Moreover, Vitamin D supplementation was positively correlated with the abundance of *Barnesiella*, as well as one unspecified family and one genus, while it was negatively correlated with the genera *Succinivibrio* and *Mitsuokella*, the family *Succinivibrionaceae*, and the order *Aeromonadales* [69].

## 4. Polyphenols

**Figure 1:**
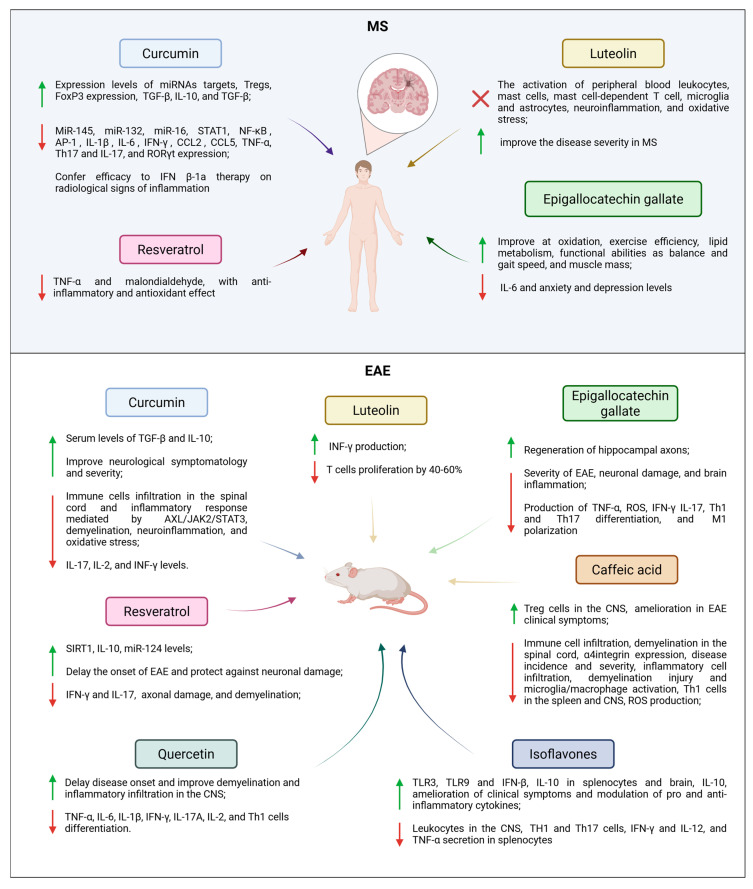
**Main effects of different polyphenols on MS and EAE.** ↑ indicates an increase or an amelioration, ↓ indicates a decrease, X indicates an inhibition. Evidence supporting the effects of polyphenols in EAE and MS includes: curcumin in EAE: [102,103,104,105]; in MS: [106,107,108,109]. Resveratrol in EAE: [110,111,112,113]; in MS: [114]. Quercetin in EAE: [115,116]. Luteolin in EAE: [117]; in MS: [118,119]. Epigallocatechin gallate in EAE: [120,121,122,123]; in MS: [124,125,126,127,128,129,130]. Caffeic acid in EAE: [131,132,133], and isoflavones in EAE: [134,135,136].

Polyphenols are a group of naturally occurring compounds known for their beneficial effects on human health and characterized by a chemical structure composed of multiple condensed phenolic cycles. They are produced through the secondary metabolism of plants and can be further classified into hydrobenzoic acids, hydroxycinnamic acids, stilbenes, lignans, and flavonoids, which in turn can be divided in flavonols, flavones, isoflavones, flavanones, anthocyanidins, and flavanols [101]. Several in-depth studies have been conducted on various polyphenols in animal models of MS, particularly focusing on curcumin, resveratrol, quercetin, luteolin, epigallocatechin gallate, caffeic acid, and isoflavones. The main effects of polyphenols on EAE and MS are summarized in Figure 1.

### 4.1. Curcumin

Curcumin is a polyphenol whose effects have been extensively studied. It is an hydroxycinnamic acid which displays several beneficial properties, as it is considered an important anticancer, antioxidant, anti-inflammatory, and antimicrobial compound which is used not only for cooking but also in the treatment of neurodegenerative disease [137]. In EAE, curcumin was found to improve neurological symptomatology and increased the serum levels of transforming growth factor β (TGF-β) and IL-10, as well as decreasing the immune cells infiltration in the spinal cord and the inflammatory response given by the microglia and mediated by Anexelekto (AXL)/Janus kinase (JAK) 2/Signal Transducer and Activator of Transcription (STAT) 3 [102,103]. Similarly, Khosropour et al. also found that the administration of both curcumin and its semisynthetic derivative F-curcumin ameliorated the onset and severity of EAE by decreasing the expression of genes encoding for IL-17, Il-2, and IFN-γ and by increasing IL-10 and TGF-β. A similar trend was also observed for the gene expression of T-cell derived transcription factors [104]. Moreover, it was found that curcumin was also able to act on toll-like receptors (TLR) by modulating their expression in EAE. Sadek et al. also demonstrated that in EAE, curcumin neuroprotective effect was controlled by adenosine monophosphate-activated protein kinase (AMPK)/sirtuin 1 (SIRT1) activation, which in turn reduced the EAE-related neuronal demyelination, neuroinflammation, and oxidative stress [105]. In pwMS, curcumin has been investigated as a potential novel therapy for its beneficial properties. One study reported that the administration for 6 months of nanocurcumin, an oral-used gel made by encapsulated curcumin in nano-micelle, to RRMS led to decrease in microRNA (miR)-145, miR-132, miR-16, STAT1, Nuclear Factor kappa-light-chain-enhancer of activated B cells (NF-κB), activator protein 1 (AP-1), IL-1β, IL-6, IFN-γ, Monocyte Chemoattractant Protein-1 (CCL2), Chemokine (C-C motif) ligand 5 (CCL5), tumor necrosis factor (TNF)-α levels in peripheral blood mononuclear cells (PBMCs) together with an increase in expression levels of miRNAs targets compared to controls [106]. Dolati et al. also investigated the regulation of dysregulated miRNA, confirming the involvement of nanocurcumin in its restoration [138]. Nanocurcumin was also demonstrated to significantly decrease Th17 and IL-17, and the expression levels of retinoid-related orphan receptor γ t (RORγt), and to increase Treg cells, FoxP3 expression, IL-10, and TGF-β in PBMCs of RRMS patients [107,108]. Lastly, a randomized controlled trial reported that supplementation with curcumin could confer efficacy to IFN β-1a therapy on radiological signs of inflammation in MS, even if it did not exert any neuroprotective effect [109].

### 4.2. Resveratrol

Resveratrol is a polyphenolic compound and a stilbene found in blueberries, red grapes, mulberries, rhubarb, pistachios, and peanuts. Due to its antioxidant and anti-inflammatory properties, resveratrol has been studied also for its neuroprotective effects [139]. Resveratrol can increase sirtuin 1 (SIRT1) levels, reduce Nicotinamide Adenine Dinucleotide Phosphate (NADPH) oxidases, NADPH Oxidase 2 (NOX2) and 4 (NOX4) levels and promote BBB integrity [110,111]. Indeed, increased SIRT1 expression has been associated with elevated levels of IL-10 and reduced levels of IFN-γ and IL-17 in EAE. Moreover, the analysis of myelin basic protein (MBP) levels and neurofilament (NF) counts demonstrated that axonal damage and demyelination are decreased upon SIRT1 overexpression [112]. It has also been shown that the pharmaceutical formulation of resveratrol, SRT501, was able to prevent neuronal damage and long-term neurological dysfunction by delaying the onset of EAE and protecting against neuronal damage [140]. Additionally, one study reported that resveratrol could improve EAE by increasing miR-124 levels, which in turn inhibit sphingosine kinase 1, leading to an amelioration in general disease outcome [113]. Regarding its antioxidant activity, Dirk et al. found with an in vitro study that resveratrol effectively modulates ROS production in MS, by increasing IL-10 levels [141]. Beneficial effects of the administration of resveratrol were observed also in a cuprizone- treated (CPZ) C57B1/6 mice model, where an amelioration in the clinical condition of the mice was registered [142]. In pwMS, resveratrol supplementation was instead found to decrease TNF-α and malondialdehyde, compared to the control, exerting an anti-inflammatory and antioxidant effect [114].

### 4.3. Quercetin

Quercetin, a flavonol found in fruits, vegetables, and certain medicinal herbs, has demonstrated anti-inflammatory and antioxidant properties in EAE and in in vitro treatments on blood cells from patients with MS [101]. It was demonstrated that quercetin was able to delay the disease onset in MS mice model and to improve the demyelination and inflammatory infiltration in the CNS. Moreover the same group demonstrated that this therapeutic action was related to the ability of quercetin to inhibit expression of proinflammatory cytokines such as TNF-α, IL-6, IL-1β, IFN-γ, IL-17A, and IL-2 [115]. A similar effect on IL-17 was also observed for quercetin pentaacetate, even if the inhibitory effect observed on Th17 cells and ROR receptor C (RORc) gene expression by quercetin was not maintained for its modified compound [143]. In CPZ MS model, quercetin significantly increased the general quality of mice ambulation, superoxide dismutase, glutathione peroxidase and total antioxidant status, while decreasing serum malondialdehyde, TNF-α, and IL-1β [144]. Similar beneficial effects were also observed in EAE, where quercetin was able to ameliorate the disease by inhibiting IL-2 signaling and Th1 cells differentiation [116].

### 4.4. Luteolin

Anti-inflammatory and neuroprotective properties were observed also for luteolin, one of the most bioactive polyphenols and flavones mainly present in fruits, vegetables, and in particular oranges, carrots, celery, broccoli, parsley, and chamomile [101]. Lutein indeed has been reported to inhibit the activation of peripheral blood leukocytes, mast cells, mast cell-dependent T cell, microglia, and astrocytes, suppress neuroinflammation and oxidative stress and improve disease severity in MS [118,119]. Moreover, it has been observed that luteolin was able to reduce the proliferation of PBMCs from pwMS and to modulate levels of IL-1β and TNF-α. At the same time, it was capable of decreasing metalloproteinase-9 (MMP-9) production and, when used in combination with IFN-β, it promoted a modulation of cell proliferation and Metallopeptidase Inhibitor 1 (TIMP-1), MMP-9, TNF-α, and IL-1β [145]. In EAE, Verbeek et al. reported that luteolin was able to reduce T cells proliferation by 40–60% compared to controls, while increasing IFN-γ production in vitro [117].

### 4.5. Epigallocatechin Gallate

Epigallocatechin gallate (EGCG) is a flavanol and the most prevalent catechin in green tea, and it is believed to be responsible for the beneficial effects of the consumption of green tea due to its several properties. Among these, it is known that consumption of green tea is associated with antioxidant effects, cancer chemoprevention, reduction of weight, improvement of cardiovascular health and protection of the skin against ionizing radiation damages [146]. EGCG was reported to reduce the clinical severity of EAE through the reduction of neuronal damage and brain inflammation, and to inhibit the production of TNF-α. Additionally, EGCG was able to block the production of reactive oxygen species in neurons [120]. Sun et al. demonstrated that EGCG inhibited IFN-γ and IL-17 production in CD4+ cells and blocked the Th1 and Th17 differentiation [121]. EGCG also exerted an effect on macrophages, by inhibiting M1 polarization, thereby promoting M2 macrophages [122]. Moreover, when used in combination with glatiramer acetate, EGCG induced regeneration of hippocampal axons in an outgrowth assay [123].

In pwMS, several clinical trials monitoring the effects of EGCG in MS have been conducted. It emerged that EGCG was able to reduce IL-6 and anxiety and depression levels, while no or low effects were reported on the EDSS, annualized relapse rate, or MRI lesion activity compared to controls [124,125]. Mähler et al. found that EGCG supplementation was also able to improve fat oxidation and exercise efficiency, and this was also confirmed by other studies in which emerged the capability of ECGC to ameliorate lipid metabolism, and functional abilities such as balance and gait speed and muscle mass [126,127,128,129,130]. Lastly, higher dosage or longer periods of EGCG supplementation were not able to lead to a reduction in brain atrophy and to significant differences in longitudinal retinal thickness measured by optical coherence tomography (OCT) between EGCG treated and placebo groups [147,148].

### 4.6. Caffeic Acid

Caffeic acid (CA) is a polyphenolic compound belonging to hydroxycinnamic acids which can be found in black chokeberries, coffee, and red wine, but that is also present in apples, plums, lingonberries, sage, thyme, oregano, marjoram, oregano, and spearmint [149]. Due to its structure, CA has several beneficial properties, as it is considered an important antioxidant with positive effects on several diseases such as obesity, diabetes, cancers, and neurodegenerative diseases [149]. These effects could be attributable to the ability of CA, as well as other polyphenolic compounds, to cross the BBB [150]. In EAE, CA phenethyl ester (CAPE) reported a reduction in immune cell infiltration, demyelination in the spinal cord and a decrease in α4integrin expression [131]. Zhou et al. analyzed instead the effect of CA phenethyl ester in MS treatment, discovering that pwMS have higher levels of proinflammatory cytokines/chemokines that preferentially skew towards T helper cytokines. In that study, it was also shown that CAPE was able to modulate T cells, to inhibit their activation and proliferation, to downregulate CD4 + IFN-γ + cells, to increase CD4 + Foxp3+, and to block NF-κB p65 nuclear translocation. Finally, Zhou et al. reported that administration of CA phenethyl ester in the EAE model led to a decreased disease incidence and severity, with less inflammatory cell infiltration, demyelination injury, and microglia/macrophage activation, and to a reduction of Th1 cells in the spleen and CNS and an increase in Treg cells in the CNS [132]. An inhibition in ROS production and an amelioration in EAE clinical symptoms was also observed in rats after the administration of CA phenethyl ester treatment [133].

### 4.7. Isoflavones

Isoflavones are phytoestrogens and a subdivision of flavonoids, which are mainly present in soy and other legumes, and which have been investigated for their anti-inflammatory and neuroprotective properties. In particular, isoflavones can suppress pro-inflammatory cytokines, enhance immune system activity and function as antioxidants [151]. Genistein, an isoflavone abundant in soy products, presents apoptotic, anti-inflammatory, and antioxidant properties, led to an amelioration of clinical symptoms and modulation of pro- and anti-inflammatory cytokines in the EAE mice model. Also, genistein treatment resulted in a reduction in rolling and adhering of leukocytes in the CNS compared to the control group [134]. Moreover, in CPZ, genistein promotes the survival of mature oligodendrocytes and myelin formation in the hippocampus [152]. All these properties exerted by genistein are probably due to its ability to modulate the factors involved in the innate immune response in the early EAE, particularly increasing toll-like receptors TLR3, TLR9, and IFN-β, IL-10 in splenocytes and the brain, and reducing Th1 and Th17 cells, IFN-γ, and IL-12 and TNF-α secretion in splenocytes [135,136]. Effects on the immune system of EAE mice were reported also for daidzein, which was able to reduce INF-γ and IL-12, increase IL-10 production, and block lymphocytes proliferation [136].

### 4.8. Polyphenols and the Gut Microbiota

Evidence highlights the ability of polyphenols to affect gut microbiota composition [153,154,155,156]. Moreover, studies report that this occurs also in EAE and MS. Jensen at al. found that the gut microbial composition in mice fed with an isoflavone-rich diet was more similar to healthy mice than those fed with an isoflavone-free diet [157]. Indeed, the gut microbiota of mice fed with an isoflavone-rich diet presented more abundant levels of *Adlercreutzia* and *Parabacteroides diastonis*, similar to healthy donors. These genera instead were depleted in pwMS, where a high abundance of *Akkermansia muciniphila* was present, as well as in the gut microbiota of mice fed with an isoflavone-free diet [157]. Additionally, curcumin administration in EAE was able to modulate the disease clinically and histologically. Moreover, curcumin administration in EAE correlated with a significant decrease in the relative abundance of *Ruminococcus bromii* and *Blautia gnavus* in feces, *Turicibacter* sp. and *Alistipes finegoldii* in ileal contents, and *Burkholderia* spp. and *Azoarcus* spp. in the ileal mucosa, all of which were associated with modulation of neuroinflammation [158]. The effects that these bacterial modulations have on MS disease have already been investigated. Bacteria like *Aldercreutzia*, which are responsible for the conversion into monomers of phytoestrogens, were reported to be reduced in RRMS compared to controls, leading to an increase of oxidative stress and inflammatory cytokines [159,160]. Therefore, an increase of *Adlercreutzia* levels after an isoflavone-rich diet could contribute to a decrease of the proinflammatory environment typically associated with MS. Similarly, *Parabacteroides distasonis* was also reported to be reduced in MS compared controls, and its increase after isoflavone administrations could be associated with beneficial and anti-inflammatory effects [161]. *Akkermansia*, a genus involved in into short-chain fatty acids (SCFAs) production and in pro-inflammatory activity, was present in higher abundance in MS patients, similar to a diet free from isoflavone, highlighting the potential anti-inflammatory role of polyphenols [162]. *Blautia gnavus*, *Turicibacter* sp., and *Alistipes finegoldii*, which were reported to be decreased after curcumin administration, are instead pro-inflammatory species associated with the presence of MS [163]. Specifically, *Turicibacter* sp. was observed to be less abundant in progressive MS compared to RRMS [164], and is associated with a 0–2 year worsening in cognition in MS [165]. Similarly, *Alistipes finegoldii* were observed to be increased in untreated MS and RRMS compared to controls [166,167]. Accordingly, curcumin may have a beneficial impact by decreasing these species’ abundance, thereby modulating the gut microbial composition toward a less pro-inflammatory profile. On the contrary, even if curcumin appeared able to reduce *Ruminococcus bromii*, previous data show that this SCFA-producing bacteria was less present in MS, and that its depletion could impair the gut barrier function and increase inflammation [168]. Lastly, *Burkholderia* spp. and *Azoarcuss* spp. are environmental pathogens, but their influence on MS development and course are still not clear. To date, only a single bioinformatic analysis has shown that a peptide sequence derived from *Burkholderia thailandensis* reaches a prediction score above 80 for CD4^+^ T-cell epitope presentation in the context of the HLA-DRB115:01 allele [169].

Even if only few studies have been conducted on the influence of specific polyphenols on the gut microbiota in EAE and MS, evidence from analysis on other diseases suggest that also other classes of polyphenols could exert beneficial influence on gut microorganisms. However, more data are still necessary.

## 5. Salt

Sodium is the main cation of the extracellular fluid (ECF) and, due to its osmotic action, is involved in the regulation of ECF volume and blood pressure. High dietary sodium intake is associated with the expansion of ECF volume and is a major risk factor for hypertension and cardiovascular disease; it also increases the risk of gastric cancer and nephrolithiasis, and promotes reduced bone mineral density and osteoporosis [170]. Salt consumption in Western diets has led to a sodium intake well above the biologically necessary and recommended limits [171]. These dietary habits not only promote the risk of cardiovascular disease but have also been hypothesized to increase the incidence of immune-mediated diseases such as MS [172]. Extracellular hyper salinity in the cytokine milieu promotes the differentiation of pathogenic Th17 cells in vitro and in vivo, and in parallel, high salt content reduces the suppressive function of Treg cells [172]. Common salt added to the diet of mice led to a severe worsening of EAE accompanied by an increase in the number of Th17 cells, an increase in the frequency of CD4+ cells expressing IL-17A in CNS infiltrates and an increase IL-17A expression in splenocytes [173]. Furthermore, in non-immunized mice, an increase in Th17 differentiation was observed in the gut and gut-associated lymphoid tissue in vivo [174]. More recently, an opposite effect of a high-salt diet was observed in an experimental murine model of spontaneous CNS autoimmune disease. It was shown how the high-salt diet increased circulating serum levels of the glucocorticoid hormone corticosterone, with the effect of enhancing the expression of tight junction molecules on brain endothelial cells and promoting the strengthening of the BBB by controlling the entry of inflammatory T cells into the CNS [175]. In a rat model with salt administration via nasogastric tube, sodium chloride was shown to produce an improvement in clinical status and a decrease in biomarkers of oxidative stress, inflammation, and dysbiosis. The mechanism by which salt causes these effects could be the renin-angiotensin-aldosterone system, which is blocked at high salt doses [176].

Data on the association between dietary sodium intake and MS risk do not seem to indicate an increased risk at higher dietary salt intake in both adult and pediatric-onset MS [177,178]. The impact of dietary sodium on the course of MS disease in humans appears to have yielded mixed results. In an initial investigation in which sodium intake was estimated by urinary excretion (single spot), a higher exacerbation rate was found in patients with medium to high sodium intake compared to the low intake group. Furthermore, individuals with high sodium intake were 3.4 times more likely to develop a new lesion on MRI and on average had eight more T2 lesions on MRI [178]. However, a subsequent investigation in which they evaluated multiple urinary sodium measurements over a 5-year follow-up in 465 newly diagnosed patients on interferon beta-1b therapy was not associated with either clinical or MRI outcomes [177].

Although salt still has an uncertain effect on MS pathology in humans, certainly the reduction of sodium intake in pwMS should be recommended for the prevention of vascular comorbidities. Hypertension appears to be more common in pwMS than in the healthy population [179]. In addition, the presence of 2 or more cardiometabolic conditions has also been associated with an increased risk of disease activity compared to the absence of cardiometabolic comorbidity. Of these cardiometabolic conditions, ischemic heart disease, hypertension, and cerebrovascular conditions were associated with MS disease activity and with worsening disability [180].

Overall, while the direct relationship between dietary sodium intake and MS remains inconclusive, current evidence highlights the importance of limiting salt consumption in pwMS, primarily to reduce comorbidities that can negatively influence disease activity and progression. A balanced sodium intake should therefore be considered an integral component of comprehensive management strategies in MS. The main evidence of salt effects in EAE and MS are summarized in Table 2.

### Salt and the Gut Microbiota

Given the reported effects of salt on the immune system, several studies have investigated whether salt intake influences the gut microbiota, to determine if microbial changes could mediate its immunological effects. Wilck et al. reported that a high-salt diet was able to affect gut microbiota composition by particularly decreasing *Ligilactobacillus murinus* (ex-*Lactobacillus murinus*) [205,206] and fecal levels of indole-3-lactic and indole-3-acetic acid metabolites. Treatment of EAE with *Ligilactobacillus murinus* and *Limosilactobacillus reuteri* (ex-*Lactobacillus reuteri*) led to an amelioration of the disease, with a reduction of Th17 cells [207]. This observed effect on EAE supports the evidence that a high-salt diet may also act in EAE by altering the abundance of these bacteria and metabolites, as bacterial supplementation led to an amelioration of the disease. However, further studies are needed to confirm these results. The impact of salt on the gut was also confirmed by another study in which salt administration was able to decrease the dysbiosis together with oxidative stress and inflammation in EAE [176]. However, although some studies have been conducted, an important gap remains in the understanding of how high salt intake affects the gut microbiota, both in EAE and MS. In particular, little is known about which microbial species are modulated, which metabolic pathways are involved, and how these changes influence the immune system. Therefore, further studies are needed, especially considering that modern dietary habits often include a high consumption of this mineral.

## 6. Essential Metals

Essential metals are elements essential for life and are contained in low concentrations in the human body. Some essential metals such as zinc, iron, and selenium play a role as cofactors in the activity of a wide range of physiological processes involved in cellular homeostasis and survival, as well as during organ and tissue development [208]. Organic concentrations of essential metals are also influenced by dietary habits [209].

Recent meta-analyses showed that serum levels of some essential metals were lower in MS patients than in healthy controls, particularly zinc, iron [186,187,188], and selenium [199,200]. The main evidence of essential metals effects in EAE and MS are summarized in Table 2.

### 6.1. Zinc

Zinc is an essential metal mainly present in food as meat, fish, and seafood, and especially in oysters, eggs, dairy products, beans, nuts, and whole grains [210]. This micronutrient appears to have a role on the immune system through an anti-inflammatory response of inhibiting the development of Th17-pathogenic lymphocytes and promoting Treg lymphocytes [211]. On the other hand, zinc can also be neurotoxic, as it is involved in neuronal death [212]. Some initial evidence seems to suggest a role for this mineral in the pathogenesis of EAE. In particular, oral administration of zinc aspartate was reported to reduce histopathological and clinical signs during the relapsing remitting phase of the disease in EAE. Together, a suppression of IFN-γ, TNF-α, Granulocyte-Macrophage Colony-Stimulating Factor (GM-CSF) and IL-5 production was observed in stimulated human T cells and mouse splenocytes, and a modulation of proinflammatory cytokines in vitro. These results aligned with previously reported data according to which injection of zinc aspartate was able to suppress EAE [181]. Contrarily, deficiency of zinc prevented the development of neurological signs of EAE in guinea pigs, with only a few cases of focal inflammatory alteration in the CNS [182]. Choi et al. found that 1H10, a zinc chelator and AMPK inhibitor, was able to reduce EAE severity and demyelination, microglial activation, immune cell infiltration, BBB disruption, and MMP-9 activation. Additionally, results show that long-term treatment with 1H10 was able to reduce the clinical course of EAE [183]. Lastly, it was reported that both zinc chelation and zinc transporter 3 (ZnT3) gene deletion significantly reduced inflammation and demyelination in the spinal cord in EAE [184,185]. Instead, PwMS were found to have higher erythrocyte zinc compared to controls [189]. On the contrary, Oraby et al. observed that disease duration, number of relapses, EDSS, and MRI lesion load were negatively correlated with zinc levels in pwMS [190]. Lastly, Cortese et al. did not observe association between dietary minerals intake as zinc and MS risk [191]. The dual nature of zinc, showing both beneficial and detrimental effects in EAE and MS, can primarily be explained by its dose-dependent behavior. Zinc must be maintained within an optimal range, as both deficiency and excess can lead to imbalance and tissue damage [213,214]. This paradox also reflects zinc’s distinct actions on the immune and nervous systems: in the immune system, zinc exerts anti-inflammatory and immunomodulatory effects [211,215,216], whereas in the central nervous system, excessive zinc accumulation can exert neurotoxic effects [217].

### 6.2. Iron

Iron (Fe) is a mineral which is introduced through the diet or through supplementation. It is an essential element found in hemoglobin, and it can be found in two main forms: heme and nonheme. Nonheme iron is contained in plants and iron-fortified foods, whereas meat, seafood, and poultry contain both heme and nonheme iron [218]. Fe is a mineral with a key role in normal neuronal processes, yet abnormal iron accumulation can cause neurodegeneration through lipid peroxidation and cell death in the brain [219]. It is possible that inadequate Fe levels, both low and high, may be detrimental in MS, as Fe reduction may decrease immune system function and cause an energy deficit due to loss of membrane potential of the mitochondria, while excess Fe may increase oxidative stress [208,219]. A study performed in 1998 on EAE revealed that mice at a clinical stage showed increased stained pathological features such as macrophages, extravasated red blood cells, and granular staining compared to the preclinical stage. Moreover, this situation persisted also in the recovery phase, but with less extravasated red blood cells [192]. Additionally, iron deficiency was reported to be protective against EAE development by Grant et al., even if iron excess was not found to be relevant for disease onset [193]. One study identified several genes associated with both MS and Fe metabolism, finding, through a Mendelian randomization analysis, a potential causal relationship between transferrin saturation and serum transferrin and MS, highlighting the link between iron metabolism and MS [220]. When considering pwMS, iron accumulation in deep grey matter was found to be strongly and independently associated with disease duration and severity [194], as well as depletion in white matter [195]. Conversely, another study reported that exacerbation of depressive disorders and a decline in quality of life of pwMS was associated with ferritin deficiency [196]. A 6-month pilot study revealed that 12 subjects taking a regimen of nutritional supplements that included also in some cases Fe supplementation, improved significantly neurologically. However, this improvement was also observed in patients who did not receive iron supplementation, suggesting that, although iron is not the only factor contributing to the beneficial effects, it does not appear to exert any negative influence on the disease [221]. Lastly, another study reported no association between dietary minerals such as iron and MS risk, either for baseline or cumulative intake during follow-up [191]. This dual effect of iron is also evident in a study conducted by Lynch et al., in which, following administration of desferrioxamine, an iron-chelating drug, one patient showed improvement, three remained stable, and five worsened by 0.5 on the EDSS scale [222]. Therefore, although the majority of studies report a detrimental role of Fe, it is likely that each patient’s condition should be evaluated individually, as different patients may respond differently to iron supplementation.

### 6.3. Selenium

Selenium (Se) is a micronutrient that can be found in edible plants that accumulate this mineral from the environment, as soil and aquatic sediments. Se is known to function as part of selenoproteins, which play an essential role in redox homeostasis, mediating synaptic plasticity and protecting CNS cells from oxidative stress and therefore the attenuation of inflammatory processes. Moreover, immunomodulatory properties of Se have been reported [223]. In EAE, it has been observed how Se reaches the CNS and reduces local inflammation and the clinical severity of the disease [197]. Additionally, another study reported that EAE animal models fed with high doses of non-toxic Se displayed higher incidence of death and developed the disease with a subacute course in some cases, compared to animals fed with lower doses of Se [198]. A first study conducted in 1988 reported lower levels of Se in MS patients compared to controls, while it was possible to obtain normal metal levels after daily Se supplementation [201]. Conversely, Alizadeh et al. did not observe differences in Se blood levels between pwMS and healthy controls [202]. In RRMS serum selenium concentrations were reported to be significantly lower than in healthy volunteers and this could also depend on dietary habits, such as higher intake of sugar, preserved products, and margarine [199]. A recent meta-analysis showed that 14.3% of MS patients use selenium as a supplement, although there is a lack of evidence regarding the safety and efficacy of selenium supplements in MS patients. However, in some randomized controlled trials, selenium-containing products were found to be well tolerated and associated with benefits with regard to inflammatory status and oxidative stress in the MS population [203]. Lastly, the administration of crocin-selenium nanoparticles, both antioxidant agents, was reported to improve the total antioxidant capacity, and the cognitive function in pwMS [204]. These findings highlight the antioxidant role of Se and suggest that the deficiencies observed in the analyzed cohorts are often attributable to inadequate dietary intake of this micronutrient. Increasing the consumption of selenium-rich foods, or considering supplementation when appropriate, may help achieve recommended intake levels and potentially exert beneficial effects in pwMS. However, further studies are needed to confirm these observations.

### 6.4. Metals and the Gut Microbiota

Essential metals such as zinc, iron, and selenium have already been described as modifiers of gut microbiota composition [224,225,226,227]. A recent study reported that high levels of arsenic, nickel, manganese, and zinc and low levels of iron, lead, titanium, and tin in MS patients compared to health controls correlated with increased abundance of *Verrumicrobiaceae*, *Lachnospiraceae*, and *Ruminococcaceae* [228]. Although a correlation has been established and it is known that these essential metals can influence gut microbiota composition, a significant gap remains in the literature regarding the effects of essential metal supplementation in EAE and MS. Further studies are therefore needed to clarify their impact on the gut microbial community.

## 7. Translational Challenges and Limitations

Despite the growing interest in nutritional interventions in multiple sclerosis, several limitations affect the interpretation and clinical translation of the available evidence.

A major limitation is the substantial heterogeneity of study designs across preclinical and clinical investigations, alongside the limited clinical validation of many compounds, as only a small proportion has been formally assessed in clinical trials. Specifically, among the vitamins, biotin emerged as one of the micronutrients most frequently investigated in clinical trials. However, the findings were inconsistent: approximately half of the cited studies reported improvements in MS-related disability [46,89], whereas the remaining trials did not observe significant benefits following Vitamin B7 supplementation [90,91]. Similarly, several clinical trials have been conducted on Vitamin D supplementation in MS patients, highlighting both positive effects, such as the reduction of disease activity in CIS and in early RRMS and amelioration on cognition [62,64], as well as an inability to reduce EDSS and the occurrence of new T2 lesions [61]. Among polyphenols, several clinical trials have examined EGCG. Most of the researches highlighted beneficial effects of ECGC, such as its ability to improve anxiety, functional capacity, muscle metabolism and percentage, gait speed, and balance and to reduce the cardiac risk and blood triglyceride levels [124,126,127,128,129]. However, some trials also failed in demonstrating a positive effect of ECGC supplementation on MS disease outcomes [125,147,148]. Lastly, among essential metals, clinical translation appears to be most feasible for iron, as iron supplementation is already widely practiced [221,222]. In contrast, for other compounds only a limited number of clinical trials in MS have been conducted or the available studies have predominantly reported molecular rather than clinical effects, as for curcumin and Vitamin B12. Consequently, further clinical trials are required to adequately assess their efficacy in the disease.

Standardized and large-scale studies are warranted to elucidate optimal dosing, bioavailability, and the potential of micronutrients and bioactive compounds in MS management. Additionally, studies highlight the importance of a balanced salt intake and appropriate dosing of essential metals, as both are fundamental to reduce the risk of comorbidities and confer beneficial effects in EAE and MS.

Another important limitation concerns dose dependency and safety. Since fat-soluble vitamins and metals can accumulate and pose toxicity risks if supplemented excessively, dose-dependency should be carefully considered in trial studies, with interventions using levels within safety limits. Overall, these limitations highlight the need for standardized, well-powered, and longitudinal clinical studies, as well as for integrative approaches that bridge mechanistic insights with clinically relevant endpoints and personalized interventions.

Personalized interventions could be developed based on the detection of nutritional biomarkers. At present, no biomarker is universally validated for guiding individualized nutrition in MS. Candidate markers under investigation include serum levels of Vitamin D, homocysteine, and markers of oxidative stress or inflammation. Low circulating Vitamin D levels have been consistently linked to increased MS risk and greater disease activity by MRI, and measuring Vitamin D status is a common clinical practice when considering supplementation strategies in people with MS. Serum 25-hydroxyvitamin D has also been associated with cognitive performance measures in MS, suggesting it may reflect broader aspects of disease biology relevant to nutritional modulation. Other potentially informative biomarkers include homocysteine, which reflects B12-vitamin status and has been associated with neuroinflammation and neurodegeneration. In any case, while these markers are biologically plausible and empirically linked to MS disease processes, their routine use to guide individualized nutritional strategies remains exploratory, pending prospective studies that directly link biomarker–guided nutrition to clinical outcomes [229].

Finally, although modulation of the gut microbiota emerges as a promising unifying mechanism, current evidence is largely associative. Causal relationships between specific microbial changes, nutritional interventions, and clinical outcomes in MS remain to be established. Dietary components such as polyphenols, vitamins, and trace elements can shape microbial composition and metabolic activity, promoting the production of bioactive metabolites, including SCFAs. These metabolites interact with the GALT and influence key immunological and molecular pathways implicated in MS. Through these mechanisms, nutrition may affect systemic inflammation, BBB integrity, oxidative stress, and neuroimmune crosstalk. Despite a chemical diversity, the action of some compounds converges on the modulation of microbial taxa involved in gut barrier integrity, SCFAs production, and immune regulation. Notably, *Akkermansia muciniphila* emerges as a recurrently modulated genus, being increased in MS, in isoflavone-free diets, following Vitamin D supplementation [68,157].

However, the currently limited evidence in this field does not yet allow the delineation of shared mechanistic frameworks underlying different micronutrient interventions in MS. Significant research gaps persist, particularly with regard to causal relationships, dose–response effects, and the clinical translation of microbiota- and pathway-targeted nutritional strategies. From this research, what appears to be clear is that future directions should emphasize personalized nutritional strategies tailored to the individual patient’s condition to maximize the potential of dietary interventions as safe and effective complementary treatments for MS.

## 8. Conclusions

MS is a chronic autoimmune and neuroinflammatory disease that affects people worldwide. While DMTs are the standard treatment for managing disease course, evidence highlights the importance of complementary lifestyle interventions, particularly a healthy and balanced diet, as an additional strategy. Therefore, this review collects together the latest findings concerning the role of vitamins, polyphenols, salt, and essential metals in MS and EAE, which are important nutritional elements the role of which is still under investigation.

These elements play a modulatory role in MS through mechanisms involving immune regulation, oxidative stress reduction, and neuroprotection. Among them, vitamins A and D appear to exert the most consistent immunomodulatory, neuroprotective, and clinical effects, whereas data regarding B, C, E, and K vitamins remain more heterogeneous. Differences among studies may reflect variations in dosage, disease stage, and study design. Nevertheless, vitamin deficiencies are frequently observed in pwMS, supporting the importance of adequate nutritional status. Polyphenols have demonstrated immunomodulatory and neuroprotective effects in EAE and, even if to a lesser extent, in clinical studies on pwMS. These effects appear to involve the regulation of pro- and anti-inflammatory cytokines, microglial activation, and blood–brain barrier integrity.

Evidence indicates that vitamins, polyphenols, dietary salt, and essential metals all modulate a set of shared immunological and molecular pathways providing valuable mechanistic insights into immune regulation, neuroinflammation, and neuroprotection.

Several compounds demonstrate a consistent ability to suppress pro-inflammatory T helper cell responses, particularly the Th17/IL-17 and Th1/IFN-γ axes, which are central drivers of MS. Vitamins A and D, curcumin, resveratrol, isoflavones, and zinc have been shown to reduce Th17 differentiation and IL-17 production while simultaneously promoting Treg expansion or IL-10 production, thereby restoring immune tolerance [58,60,77,78,79,102,103,104,107,108,112,135,136,141,211]. Moreover, Vitamins B1 and B5, quercetin and EGCG were able also to decrease the Th17 and IL-17 production [30,39,115,121,143], while Vitamin C and CA promoted Treg differentiation and IL-10 production [25,132]. Conversely, salt was reported to exert an opposite effect, by favoring Th17 and IL-17 expansion and decreasing Treg proliferation [172,173].

A prominent feature of many compounds is the downregulation of pro-inflammatory cytokines such as IL-6, IL-1β, TNF-α, and IL-2. Vitamins A, B2, B6, C, D, curcumin, resveratrol, quercetin, luteolin, EGCG, isoflavones, and zinc inhibit these pro-inflammatory cytokine production, also through suppression of NF-κB signaling, a master regulator of inflammatory gene expression [25,34,35,42,58,104,106,114,115,124,125,135,136,144,145,181]. In parallel, antioxidant activity, particularly via reduction of ROS and iNOS expression, contributes to the decreased of oxidative stress within both peripheral immune cells and CNS-resident glia, mainly driven by Vitamins A, B6, C, E, polyphenols, selenium and, according some studies, also to NaCl [25,42,95,97,101,114,118,119,134,137,139,141,144,146,149,151,176,204].

Activation of the Nrf2/HO-1 pathway emerges as a shared protective mechanism among antioxidants such as Vitamins A and C [25]. Nrf2 activation enhances cellular antioxidant defences and limits microglial overactivation, which is a critical contributor to neurodegeneration and demyelination. Accordingly, some compounds in the table are associated with reduced microglial activation, as Vitamins C, CA, and luteolin [25,118,119,132].

Importantly, some molecules exhibit direct neuroprotective and regenerative effects. Vitamin B3 and Vitamin E have been linked to oligodendrocyte survival and proliferation, supporting remyelination processes [37,70]. In addition, Vitamins B1, C, curcumin, quercetin, luteolin, CA, and NaCl reduce CNS immune cell infiltration, likely by stabilizing the BBB and downregulating MMP9 [25,30,102,103,115,131,132,145,173]. The opposite effect was instead observed for zinc, which promoted CNS infiltration.

**Figure 2:**
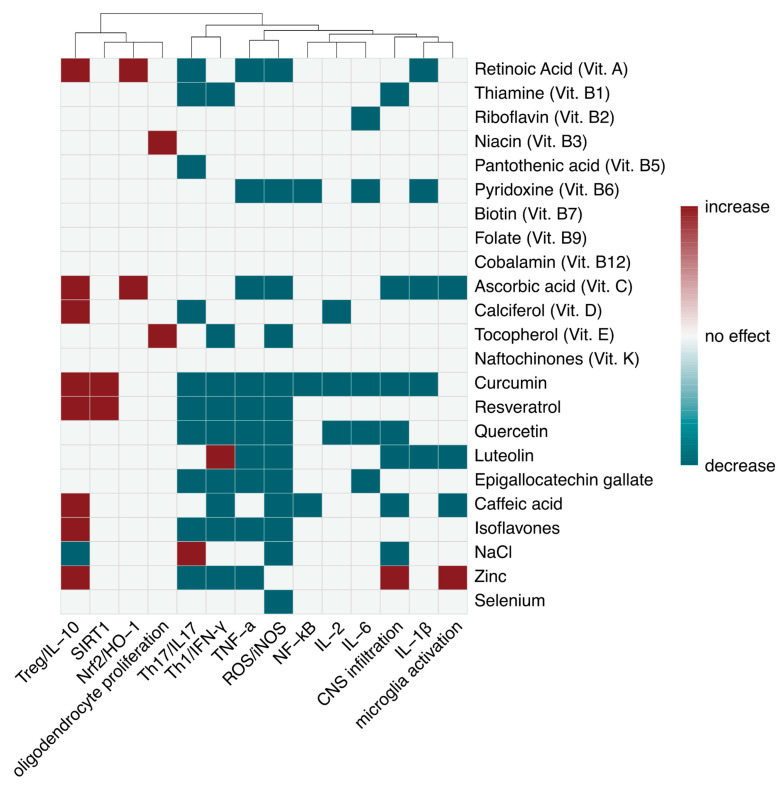
**Conceptual heatmap of neuroinflammatory effects of vitamins, polyphenols, salt and essential metals in MS/EAE.** Supporting evidence is included in the text above.

Overall, the convergence of these compounds on shared inflammatory and redox-regulatory pathways suggests that targeting common nodes may be more effective than focusing on single molecules or isolated mechanisms. This overlap supports the rationale for multimodal nutritional or nutraceutical strategies, potentially combining vitamins, polyphenols, salt, and essential metals to achieve synergistic effects on immune regulation and neuroprotection. Such pathway-oriented interventions could represent accessible, low-risk adjunct approaches to conventional therapies aimed at preventing or attenuating CNS inflammatory and autoimmune diseases. Immunological and molecular pathways shared by Vitamins, polyphenols, salt, and essential metals are represented in Figure 2.

In conclusion, the clinical translation of these findings remains limited, as human trials often yield heterogeneous outcomes, and further investigations in pwMS are warranted. Indeed, while the evidence from EAE models is often promising, clinical application is not yet supported by sufficient evidence, as only a few studies have been performed so far and they are mostly contradictory or inconclusive or lack human supplementation trials entirely.

Given the complex interplay between nutrition, immune regulation, and neuroinflammation, a deeper understanding of the roles of vitamins, polyphenols, salt, and essential metals in MS is essential. Indeed, this will enable the development of safe, accessible, and personalized dietary interventions capable of supporting conventional therapies, slowing disease progression, and improving the quality of life of MS patients.

## Figures and Tables

**Table 1:** Effects of vitamins on EAE and MS.

Vitamin	Effects in EAE	Effects in MS
**A (Retinol)**	Suppression of EAE [24]; Co-supplementation with Vitamin C mitigates neurological severity and disease progression [25].	↑ the B cells-secreted IL-10 [26]; ↓ and prediction of the risk of developing gadolinium-enhancing T1 lesions, T2 lesions, and active lesions [27]; Improve MSFC in RRMS [28]; Improve fatigue and depression and psychiatric outcomes [29].
**B1 (Thiamine)**	↓ of disease progression, acceleration of its onset and microglial activation [30]; ↓ in Th1 and Th17 cell infiltration in the spinal cord, with amplification of the inflammatory response [30].	↓ depression [31]; ↓ of fatigue [32]; Improvement in gut dysbiosis by B1, B2, B3, B5, B6, and B12 together [33].
**B2 (Riboflavin)**	Amelioration of neurological disability mediated by BDNF and IL-6 [34]; Synergistic effects with IFN-β1a [35].	No improvement in EDSS [36]; Improvement in gut dysbiosis by B1, B2, B3, B5, B6, and B12 together [33].
**B3 (Niacin)**	↑ neurological functional recovery [37]; ↓ of inflammatory infiltrates [37]; ↑ in oligodendrogenesis and axonal regeneration [37]; No effect on EAE clinical score without amelioration of neuropathology [38]; No ↓ in T cell proliferation [38].	Improvement in gut dysbiosis by B1, B2, B3, B5, B6, and B12 together [33].
**B5** **(Panthotenic acid)**	Amelioration of Th17-associated autoimmune disorders [39].	↑ in the serum of MS patients [40]; ↓ in MS patients [41]; Improvement in gut dysbiosis by B1, B2, B3, B5, B6, and B12 together [33].
**B6 (Pyridoxine)**	↓ of sphingosine-1-phosphate [42]; Prevention of EAE development [42].	Low B6 levels were associated with higher EDSS [43]; Improvement in gut dysbiosis by B1, B2, B3, B5, B6, and B12 together [33].
**B7 (Biotin)**	B7 deficiency ↓ thymus size, cellularity, and immune response [44];	Improvement in 25-foot walk time [45]; Impact on disability and disease progression [46].
**B9 (Folate)**		No significant differences in B9 concentrations between pwMS and healthy controls [47]; Improvement in the mental and physical fields of quality of life when combined with B12 [48].
**B12 (Cobalamin)**	Improvement in clinical motility with IFN- beta therapy and ↓ astrocytosis and demyelination [49];	No significant differences in B9 concentrations between pwMS and healthy controls [47]; B12 deficiency is not associated with MS [50]; Lower levels of B12 both in the serum and in the cerebrospinal fluid ↑ earlier age of MS onset [48,51,52]; Improvement in the mental and physical fields of quality of life when combined with B12 [48]; Improvement in gut dysbiosis by B1, B2, B3, B5, B6, and B12 together [33].
**C (Ascorbic acid)**	No effect on EAE, only moderate effects on the development of clinical symptoms and no prevention in the opening of the BBB [53]; Mitigation of neurological severity and disease progression, ↓ in demyelination, inflammation, immune cell infiltration, and activation of microglia and astrocytes [25].	Lower levels during a relapse and with infratentorial lesions [54,55]; No ↓ in the risk of MS onset [56].
**D (Calciferol)**	EAE development require Vitamin D and its receptors [57]; Inhibition of Treg and Th17 differentiation and migration [58].	↓ of MS incidence [59]; ↓ risk of developing MS [59]; ↑ IL-10 levels [60]; No ↓ in EDSS scores or new T2 lesions [61]; ↓ disease activity in CIS and in early RRMS [62]; Low Vitamin D worsen information processing speed performances in MS [63]; ↑ cognition [64]; Disruption of intestinal barrier integrity, dysbiosis and reduction in butyrate-producing bacteria caused by Vitamin D deficiency [65,66,67]; ↑ *Faecalibacterium*, *Coprococcus*, *Akkermansia* and *Barnesiella* [68,69]; ↓ *Succinivibrio*, *Mitsuokella*, *Succinivibrionaceae* and *Aeromonadales* [69].
**E (Tocopherol)**	↑ oligodendrocyte regeneration and remyelination [70]; ↓ EAE severity and delay disease progression by suppressing T cell proliferation and the Th1 immune response [71].	Lower vitamin E in MS serum [54]; ↓ in lipid peroxidation in serum and maintain telomere length in circulating lymphocytes [72]; No ↓ in the risk of MS onset [56].
**K (Naphthoquinones)**	Improvement in disease outcome [73].	Low Vitamin K levels in MS [74].

↑ indicates an increase, ↓ indicates a decrease.

**Table 2:** Effects of salt and essential metals on EAE and MS.

Compound	Effects in EAE	Effects in MS
Salt (NaCl)	↑ Th17 differentiation [172,173,174]; ↓ Treg suppressive function under high-salt conditions [172]; Worsening of EAE severity [173]; ↑ IL-17A^+^ CD4^+^ CNS infiltrates and splenocyte IL-17A expression [173]; ↑ corticosterone levels with strengthening of BBB integrity [175]; ↓ CNS T cell infiltration in spontaneous CNS autoimmunity [175]; ↓ oxidative stress, inflammation, and dysbiosis [176].	No clear association between high sodium intake and MS risk in adults and pediatric-onset MS [177,178]; ↑ relapse rate and MRI activity [178]; No association with clinical or MRI outcomes [177];
Zinc	↓ clinical and histopathological EAE severity [181]; ↓ IFN-γ, TNF-α, GM-CSF, IL-5 [181]; Zinc deficiency prevents neurological signs of EAE [182]; Zinc chelation or ZnT3 deletion ↓ inflammation, demyelination, BBB disruption, and microglial activation [183,184,185].	↓ serum zinc levels in pwMS compared to controls [186,187,188]; ↑ erythrocyte zinc levels in pwMS [189]; ↓ zinc levels associated with ↑ disease duration, relapse number, EDSS, and MRI lesion load [190]; No association between dietary zinc intake and MS risk [191].
Iron	↑ macrophage infiltration, extravasated red blood cells and granular staining during clinical EAE [192]; Iron deficiency protective against EAE development [193]; Iron excess not clearly associated with disease onset [193].	↓ serum iron levels in pwMS [186,187,188]; ↑ iron accumulation in deep grey matter associated with disease duration and severity [194]; ↓ iron levels in white matter [195]; Ferritin deficiency associated with depressive symptoms and reduced quality of life [196]; No association between dietary iron intake and MS risk [191].
Selenium	↓ local inflammation [197]; ↓ clinical severity of EAE [197]; ↑ mortality and subacute disease course in EAE [198].	↓ serum selenium levels in pwMS in several cohorts [199,200,201]; No differences in Se levels between pwMS and controls in some studies [202]; Selenium supplementation associated with ↓ inflammatory and oxidative stress markers [203]; Crocin–selenium nanoparticles ↑ antioxidant capacity and cognitive function [204].

↑ indicates an increase, ↓ indicates a decrease.

## Data Availability

This review is based on previously published studies. No new data were generated or analyzed.
